# Case report: Treatment of two cases of recurrent/refractory early T-cell precursor acute lymphoblastic leukemia with venetoclax combined with the CAG regimen

**DOI:** 10.3389/fmed.2024.1358161

**Published:** 2024-03-08

**Authors:** Yuxia Jiang, Lin Ji, Xin Jin, Haiying Wu, Mingxia He, Fenglin Shen, Xiaofeng Xu, Huifang Jiang

**Affiliations:** ^1^Tongde Hospital of Zhejiang Province, Hangzhou, Zhejiang, China; ^2^Hangzhou Red Cross Hospital, Hangzhou, Zhejiang, China

**Keywords:** venetoclax, CAG, recurrent/refractory, early T-cell precursor acute lymphoblastic leukemia, HSCT

## Abstract

Early T-cell precursor acute lymphoblastic leukemia (ETP-ALL) is a highly aggressive subtype of T-ALL. No standard chemotherapy regimen exists for patients with recurrent/refractory (R/R) ETP-ALL; in these patients, the primary goal of salvage therapy is to achieve remission as a foundation for consolidation and intensification treatments. This study reports cases of two patients with R/R ETP-ALL who underwent salvage therapy of venetoclax combined with the CAG regimen and achieved complete remission in the bone marrow. Flow cytometry results were negative for minimal residual disease. Both patients were bridged to allogeneic hematopoietic stem cell transplantation (HSCT) and in complete remission over a 3-year follow-up period. These cases show that the use of venetoclax combined with the CAG regimen may offer patients with R/R ETP-ALL an opportunity for allogeneic HSCT.

## Introduction

EarlyT-cell precursor acute lymphoblastic leukemia (ETP-ALL) is a highly aggressive subtype of T-ALL characterized by the abnormal expression of myeloid/stem cell antigens. ETP-ALL responds poorly to conventional induction chemotherapy and has a lower remission rate, higher recurrence rate, and worse prognosis than other types of T-ALL (1–5). Despite the availability of new targeted therapies, the remission rate in patients with recurrent/refractory (R/R) ETP-ALL remains below 18% (1, 6). In this report, we present two cases of adult patients with R/R ETP-ALL who underwent salvage therapy with a combination of venetoclax and the CAG (cytarabine 10 mg/m^2^ q12h d1–14 + aclarubicin 20 mg d1–4 + granulocyte colony stimulating factor (G-CSF) 200 μg/m^2^ qd d1–14) regimen. The regimen was well-tolerated and the patients were bridged to allogeneic peripheral blood hematopoietic stem cell transplantation (HSCT). The patients were considered cured after 3 years of follow-up.

## Case presentation

### Case 1

**FIGURE 1:**
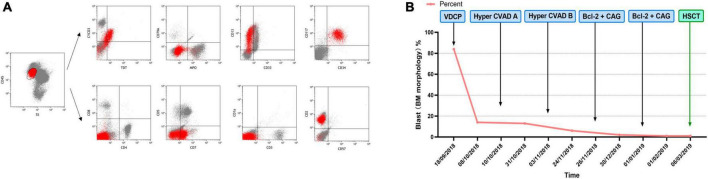
**(A)** Immunophenotype of Case 1 from bone marrow flow cytometry. **(B)** Chemotherapy regimen and therapeutic outcome of Case 1.

A 42-year-old male who was diagnosed with T-ALL in August 2018 presented with recurrent fever. He had been undergoing chemotherapy with the VDCP (vincristine 2 mg d1, 8, 15, 22 + daunorubicin 68 mg d1–3, 15 + cyclophosphamide 1.2 g d1, 15 + prednisone 65 mg d1–7, 45 mg d15–28) and hyper-CVAD A/B (cyclophosphamide 540 mg q12h d1–3 + doxorubicin 90 mg d4 + vincristine 2 mg d4, 11 + dexamethasone 40 mg d1–4, 11–14 / methotrexate 2 g d1 + cytarabine 1 g q12h d2–3) regimens but failed to achieve remission. Complete blood count on October 31, 2018: white blood cell (WBC) count, 2.6 × 10^9^/L; hemoglobin (Hb), 86 g/L; and platelet (PLT) count, 62 × 10^9^/L. Bone marrow morphology suggested 6% lymphoblastoid cells with negative MPO staining. Bone marrow flow cytometry indicated 5.26% abnormal T-lymphoblasts. Immunophenotyping indicated that these abnormal cells expressed TdT, CD13, CD7, CD117, CD34, CyCD3, and CD2 but not CD4, CD5, CD8, CD3, CD1a, MPO, CD79a, CD33, and CD57 (Figure 1A). Cytogenetics indicated a normal karyotype. Next-generation sequencing indicated mutations in ARID2, KMT2D (MLL2), DNMT3A, IDH2, and TET2. After reviewing the results of bone marrow flow cytometry at the time of the patient’s initial consultation, it was determined that a diagnosis of ETP-ALL should have been made at that time. Thus, the patient’s diagnosis was revised to R/R ETP-ALL in light of his medical history. On November 26, 2018, the patient commenced treatment with venetoclax and the CAG regimen (venetoclax 100 mg d1, 200 mg d2, 400 mg d3–28 + cytarabine 25 mg q12h d1–14 + aclarubicin 20 mg d1–4 + G-CSF 300 μg qd d1–14). Body temperature returned to normal after chemotherapy and bone marrow flow cytometry performed 35 days after the initiation of venetoclax and the CAG regimen was negative for minimal residual disease (MRD). On January 1, 2019, the patient again received venetoclax and the CAG regimen, and the bone marrow remained negative for MRD. On March 6, 2019, the patient underwent successful child-to-parent haploidentical peripheral blood allogeneic HSCT. Over a 3-year follow-up period, the patient’s bone marrow consistently tested negative for MRD (Figure 1B).

### Case 2

**FIGURE 2:**
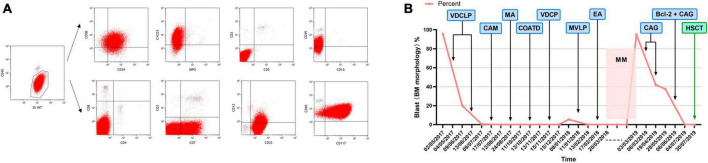
**(A)** Immunophenotype of Case 2 from bone marrow flow cytometry. **(B)** Chemotherapy regimen and therapeutic outcome of Case 2.

A 25-year-old male was diagnosed with ETP-ALL in May 2017. Immunophenotyping indicated that these abnormal cells expressed CD33, CD13, CD117, CD34, CD7, and CyCD3 but not CD4, CD8, CD1a, and CD5 was used to diagnose ETP-ALL. Bone marrow complete remission (CR) was achieved after two standard cycles of the VDCLP (vincristine 2 mg d1, 8, 15, 22 + daunorubicin 76 mg d1–3, 15, 16 + cyclophosphamide 1.425 g d1, 15 + L-asparaginase 10000 IU d11, 14, 17, 20, 23, 26 + prednisone 70 mg d1–7 50 mg d15–28) chemotherapy regimen, which started on May 4, 2017. The patient successively received the MA (mitoxantrone 11.4 mg d1–3 + cytarabine 700 mg q12h d1–3), CAM (cyclophosphamide 1.425 g d1 + cytarabine 95.5 mg q12h d1–3, 8–10 + 6-mercaptopurine 110 mg d1–7), COATD (idarubicin 10 mg d1 5 mg d2 + cyclophosphamide 1.425 g d1 + vindesine 4 mg d1 + dexamethasone 10 mg d1–7 + cytarabine 95.5 mg q12h d1–3), and VDLP (vincristine 2 mg d1, 8, 15, 22 + daunorubicin 76 mg d1–3 + L-asparaginase 10000 IU d11, 14, 17, 20, 23, 26 + dexamethasone 15 mg d1–7, 10 mg d15–21) regimens starting on June 13, 2017, during which CR was achieved on bone marrow re-examination. However, a complete blood count taken on January 8, 2018, showed: WBC, 3.2 × 10^9^/L; Hb, 83 g/L; and PLT, 46 × 10^9^/L. Bone marrow morphology re-examination showed 5.5% pre/pro-lymphocytes and bone marrow flow cytometry indicated that the immunophenotype was consistent with the initial onset, suggestive of leukemia recurrence. On January 11, 2018, the patient received one cycle of MVLP (methotrexate 5.5 g dl + vindesine 4 mg d2 + dexamethasone 10 mg d1–5 + L-asparaginase 10000 IU d3–4) and EA (etoposide 0.1 g d1–3 + cytarabine 90 mg q12h d1–4) regimens, which resulted in a negative MRD bone marrow test. On February 23, 2018, maintenance therapy with the MM regimen (6-MP 100 mg qd + MTX 30 mg qw) was initiated. Recurrence occurred 14 months after bone marrow CR was achieved. Complete blood count in March 2019 showed: WBC, 1.2 × 10^9^/L; Hb, 93 g/L; and PLT, 16 × 10^9^/L. Bone marrow morphology suggested 95% lymphoblastoid cells with negative MPO staining and flow cytometry indicated 84% abnormal T-lymphoblasts. Immunophenotyping indicated that the abnormal cells expressed CD58, CD34, CD7, CD117, CD33, CD13, and CyCD3 but not MPO, CD5, CD2, CD1a, CD4, CD8, and CD3 (Figure 2A). Cytogenetics indicated a normal karyotype and next-generation sequencing indicated mutation in JAK3. Therefore, the patient was diagnosed with R/R ETP-ALL. The patient was then treated with two cycles of the CAG regimen (aclarubicin 20 mg qd d1–4 + cytarabine 37 mg q12h d1–7 + G-CSF 300 μg qd d1–14) starting on March 6, 2019, and bone marrow remission was not achieved. On June 6, 2019, the patient received venetoclax plus the CAG regimen (venetoclax 100 mg d1, 200 mg d2, and 400 mg qd d3–28 + aclarubicin 20 mg d1–4 + cytarabine 37.4 mg qd d1–10 + G-CSF 300 μg qd d1–8), and bone marrow was found to be negative for MRD. On July 30, 2019, the patient successfully underwent parent-to-child allogeneic peripheral blood HSCT. Over a 3-year follow-up period, the patient’s bone marrow remained negative for MRD (Figure 2B).

## Discussion

ETP-ALL is a novel, high-risk variant of ALL characterized by poorer clinical outcomes and higher rates of drug resistance and recurrence than typical T-ALL; it has a 10-year cumulative incidence of remission failure or hematologic relapse of 72% compared with typical T-ALL (1, 5). Although there is no significant difference in overall survival between children with ETP-ALL and those with T-ALL (86 vs. 90%) (7), the complete remission rate of adults with ETP-ALL is significantly lower than that in those with non-ETP-ALL, and the median overall survival of patients with ETP-ALL is only 20 months (5).

The diagnosis of ETP-ALL is based on a combination of immunophenotypic characterization and gene expression patterns. (1) Immunophenotype: positive for CD7, CD2, cytoplasmic CD3, and CD4; generally negative or < 75% positive for CD5; positive for one or more of the myeloid/stem cell antigens CD34, CD117, HLA-DR, CD13, CD33, CD11b, or CD65; and negative for CD1a and CD8 (8). (2) Molecular genetics: often accompanied by mutations in myeloid-associated genes such as FLT3, NRAS/KRAS, DNMT3A, IDH1, and IDH2; mutations common in T-ALL such as in NOTCH1 and CDKN1/2 are rare (9, 10). ETP-ALL is often misdiagnosed owing to the atypical clinical presentation, difficulty in cytologic diagnosis, and insufficient knowledge of flow cytometric and molecular genetic features. Case 1 was initially diagnosed with T-ALL but conventional standard VDCP and hyper-CVAD AB regimens were ineffective. The bone marrow was re-examined and immunophenotyping by flow cytometry revealed obvious myeloid and stem cell markers, including CD13, CD33, CD34, CD7, and CYCD3. T cell markers were positive for CD7 and CyCD3 but negative for CD5, CD4, CD8, and CD1a. When combined with next-generation sequencing results, which revealed multiple myeloid tumor-associated mutations, the final diagnosis was revised to R/R ETP-ALL.

ETP-ALL is often misdiagnosed owing to a lack of awareness of ETP-ALL among clinicians. In such patients, conventional combination chemotherapy is ineffective or effective but prone to recurrence and refractory disease, leading to the early emergence of R/R ETP-ALL at the time of clinical treatment. Case 1 did not achieve remission after chemotherapy with the standard VDCP and hyper-CVAD A/B regimens. Case 2 achieved remission with a conventional chemotherapy regimen for ALL but developed recurrence twice. No standard chemotherapy regimen exists for R/R ETP-ALL; thus, researchers have investigated novel treatment approaches. ETP-ALL has been hypothesized to have a gene expression profile similar to that of hematopoietic stem cells and bone marrow progenitors owing to its arrest at an early stage of T-cell differentiation and myeloid differentiation potential, with high expression of genes associated with self-renewal, including LMO2, LYL1, and HHEX, as well as anti-apoptotic BCL-2 (11). Other studies have demonstrated better response with acute myeloid leukemia (AML)-type regimens, such as conventional doses of cytarabine plus 6-mercaptopurine and cyclophosphamide (12, 13). These regimens have shown better response rates than ALL-type regimens; however, the results are still deemed unsatisfactory. In Case 2, ETP-ALL similarly progressed to R/R ETP-ALL without satisfactory results following treatment with the CAG regimen. Thus, the treatment of R/R ETP-ALL has proven challenging to clinicians.

Anti-apoptotic BCL-2 family proteins mediate clonal selection and survival, advantage with important roles in lymphocytes. BCL-2 protein expression is higher in ETP-ALL than in non-ETP-ALL. Venetoclax, when administered alone, alters the proliferation of human T-ALL cell lines and primary cells, particularly those with an ETP phenotype (14, 15). A strong synergistic effect between venetoclax and cytarabine has also been reported (15, 16). Therefore, we explored the use of venetoclax in conjunction with the CAG regimen for the treatment of R/R ETP-ALL. Complete remission was achieved in both patients with the venetoclax + CAG regimen with safe and manageable adverse effects, and ultimately, long-term survival in conjunction with allogeneic HSCT.

In conclusion, two individuals with R/R ETP-ALL successfully went into remission using venetoclax plus the CAG regimen, which may represent a novel and effective option for the treatment of patients with R/R ETP-ALL.

## Data availability statement

The original contributions presented in the study are included in the article/supplementary material, further inquiries can be directed to the corresponding authors.

## Ethics statement

This case report has been granted an exemption from ethics Review Committee of Zhejiang Litongde Hospital: NO. 140-JY 2022. The studies were conducted in accordance with the local legislation and institutional requirements. The participants provided their written informed consent to participate in this study. Written informed consent was obtained from the individual(s) for the publication of any potentially identifiable images or data included in this article.

## Author contributions

YJ: Writing – original draft, Writing – review and editing. LJ: Writing – original draft. XJ: Writing – original draft, Data curation. HW: Writing – original draft. MH: Writing – original draft. FS: Writing – original draft. XX: Writing – original draft, Writing – review and editing. HJ: Writing – original draft, Writing – review and editing.
